# Outcomes of inpatient psychological treatments for children and adolescents with eating disorders at time of discharge: a systematic review

**DOI:** 10.1186/s40337-020-00307-2

**Published:** 2020-07-03

**Authors:** Leanna Isserlin, Wendy Spettigue, Mark Norris, Jennifer Couturier

**Affiliations:** 1 Department of Psychiatry, Children’s Hospital of Eastern Ontario, University of Ottawa, Ottawa, ON K1H 8L1 Canada; 2 Children’s Hospital of Eastern Ontario Research Institute, Ottawa, ON K1H 8L1 Canada; 3 Division of Adolescent Health, Department of Pediatrics, Children’s Hospital of Eastern Ontario, University of Ottawa, Ottawa, ON K1H 8L1 Canada; 4 Department of Psychiatry & Behavioural Neurosciences, McMaster University, Hamilton, ON L8N3Z5 Canada

**Keywords:** Child, Adolescent, Humans, Inpatients, Hospitalization, Feeding and eating disorders

## Abstract

**Background:**

Recommended first line treatment for children and adolescent eating disorders is outpatient therapy. However, a significant number of children and adolescents with eating disorders continue to require inpatient treatment during the course of their illness. The effect of psychological treatments in an inpatient setting on outcomes at the time of discharge remains unclear. This paper presents the results of a review of the literature on outcomes at the time of discharge following inpatient psychological treatment for children and adolescents with eating disorders.

**Main body:**

The majority of studies found were observational and of low quality. The most consistently reported positive outcome of inpatient treatment is weight gain. Results related to symptom change and motivation vary between studies. Within the inpatient setting, there is considerable heterogeneity in the types of treatments offered, goals of treatment, length of stay and outcomes measured.

**Conclusion:**

There remains a paucity of high-quality studies examining the effect of psychological treatments provided to children and adolescents in an inpatient setting. The significant heterogeneity between studies makes it not possible to compare across studies. Future research should aim to resolve these deficiencies in order to better determine the specific factors that contribute to positive outcomes of inpatient treatment for children and adolescents with eating disorders.

## Plain English summary

For children and adolescents with eating disorders, outpatient treatment is the type of treatment with the most support in the scientific literature and should be the basis of most treatment plans. However, many children and adolescents will require inpatient treatment at some point in their illness. The effect of inpatient treatment on eating disorder symptoms remains unclear. This paper aims to review the available literature on psychological treatments provided to children and adolescents with eating disorders in an inpatient setting in order to understand what is known to date. Unfortunately, there remain very few studies designed in such a way to be able to answer the question ‘what is the best psychological treatment to provide in an inpatient setting to children and adolescents with eating disorders?’ and due to considerable variability in the studies available, the only consistent positive result in the studies available is that inpatient treatment is effective in producing weight gain. Further research is needed in order to optimize treatment for children and adolescents with eating disorders in the inpatient setting.

## Background

Eating disorders (EDs) are complex disorders that typically appear during childhood and adolescence [1]. Current ED guidelines and expert consensus support outpatient family-based treatment as the first-line recommended treatment for children and adolescents [2–4]. Despite this recommendation, a proportion of patients with potentially life-threatening severe EDs will require higher levels of support, including inpatient stabilization and treatment [5].

Unfortunately, inpatient lengths of stay (LOS) for ED treatment tend to be long in comparison to other medical and psychiatric disorders, with the average LOS in studies from Germany, Japan, Switzerland and Scotland being greater than 7 weeks [6–9]. Modifying factors for LOS in adolescent patients include treatment under the Mental Health Act and nasogastric feeding, both of which appear to be associated with longer LOS [9]. Prolonged LOS is concerning, as it can disrupt adolescent development and engagement in school, family and social life. Further, inpatient treatment is expensive in terms of economic costs and intensive medical resource requirement [10]. In 2013, a review of admissions with a mean length of stay 37.9 days (standard deviation [SD] 19.7 days) to a Canadian tertiary care center for medical stabilization and initial weight rehabilitation for adolescents with AN found that the mean total hospital cost was $51,349 Canadian Dollars (CAD) (SD $26598) with a mean total societal cost of $54,932 CAD (SD $27864) per admission [10]. Similar studies from Portugal and Germany found comparable high costs for eating disorders treatment, in that the average cost per admission in those countries was 5202 to 5883€ and 13,367€ respectively [11, 12].

While three recently published studies examining outcomes across varying treatment settings supports the least intensive management as first line treatment for EDs [13–15], the evidence base that examines best practice for patients who do require inpatient treatment remains scant. The goal of this review is to further explore the scope and benefits of psychological treatments provided to children and adolescents with EDs in inpatient settings at the time of hospital discharge.

## Methods

PRISMA systematic review methodology was used to capture all articles on inpatient treatment for EDs in children and adolescents [16]. Our search utilized the following databases: Medline, PsycINFO, EMBASE, Cochrane Database of Systematic Reviews, Cochrane Central Register of Controlled Trials (CENTRAL) and CINAHL, and included the following search terms: Anorexia Nervosa (AN) OR Bulimia Nervosa (BN) OR Binge Eating Disorder (BED) OR Other Specified Feeding and Eating Disorder (OSFED) OR Eating Disorder Not Otherwise Specified (EDNOS) OR Avoidant/Restrictive Food Intake Disorder (ARFID) AND Inpatient Treatment. The search string was developed further and was modified for each database as appropriate (see Additional file 1). Inpatient treatment was defined for the purposes of this review as treatment that involves 24 h/day care in a hospital setting. Reference lists were reviewed for any additional articles. Two reviewers had to agree for inclusion of articles in our review, with a third reviewer resolving any disputes. The initial database search included all years up until March 2017. In November 2018 a forward citation chaining process was completed to search each included article to examine if it had been cited by any additional articles since March 2017 up until November 2018. Newly found articles were then screened to decide whether they matched the inclusion criteria. The forward chaining process involved the use of Google Scholar to locate all articles citing our included articles from the primary search. Inclusion criteria included original peer-reviewed research articles that focused on children and adolescents up to age 18 years, provided a description of an ED-specific treatment as well as weight and/or psychological outcomes at the time of discharge, and were published in English or French. Studies of all different types of methodology were included (randomized controlled trials [RCTs], open trials, case reports). Studies were excluded if they did not distinguish between outcomes for children and adolescents and outcomes for adults, or if they focused only on medical interventions, pharmacotherapy or refeeding protocols aimed at restoring medical stability to facilitate discharge to outpatient treatment.

For each included study, the study type (e.g. prospective or randomised control trial), number of participants included in the analysis, treatment method, primary outcome variable(s), and observed results were summarized.

Given the low quality of the studies, quantitative analysis was not possible. Qualitative review of all included studies was performed, summarizing the outcomes at end of inpatient treatment. We distinguish outcomes across various types of treatment frameworks as well as the evidence supporting the use of adjunctive therapies as part of inpatient treatment.

Two reviewers (LI and JC) independently assessed the risk for bias in the articles using the GRADE (Grading of Recommendations, Assessment, Development and Evaluations) system [17]. The GRADE system incorporates analysis of risk of bias based on various limitations of the studies, including lack of concealment of allocation, blinding, accounting of all participants, and selective reporting of outcomes for RCTs, and lack of an adequate control population, flawed measurement of outcomes, failure to adequately control for confounding factors and incomplete or inadequately short follow-up for observational studies.

## Results

**Fig. 1:**
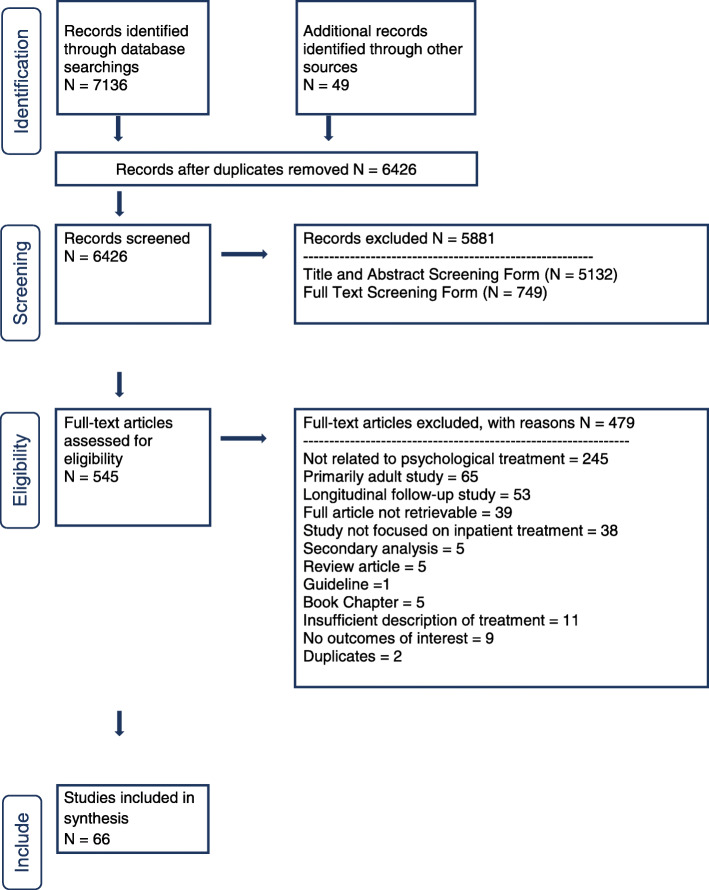
PRISMA (Preferred Reporting Items for Systematic Reviews and Meta-Analyses) flow diagram of study selection

The database search initially provided *n* = 7136 citations, as reported in the PRISMA flowchart (Fig. 1). An additional 49 citations were added through review of references and forward citation chaining. After removing duplicates, *n* = 6426 records remained, of which *n* = 5881 were eliminated given that they did not meet the inclusion criteria. Of the 545 full text articles assessed for eligibility, *n* = 479 studies were excluded because they were longitudinal follow-up studies with no information on outcome at the time of discharge, were primarily adult studies, were review or secondary analysis papers, book chapters or guidelines, or did not provide sufficient description of the treatment provided, did not focus on inpatient treatment, or otherwise did not meet the inclusion criteria. Ultimately, *n* = 66 studies were selected for inclusion in this review (See Fig. 1). Quantitative analysis of the results was not possible due to the varying outcome measures used, the low quality of the studies and the high heterogeneity in methods used amongst the studies. Instead a narrative summary of the results is presented below. The risk of bias as assessed by the GRADE system was deemed as high for all included studies.

### Inpatient ED programs for anorexia nervosa

#### Primary outcome – weight gain

We failed to identify any RCTs exploring outcomes of inpatient treatment specifically for pediatric AN, including atypical anorexia nervosa. We identified twenty studies (total *n* = 1091) describing inpatient treatment based on an integrative approach for AN, including psychiatric, medical, nutritional, and pharmacologic management, as well as individual, group and family therapy along with skilled nursing support [18–37]. None of these studies included control or comparison groups. Various measures of change in weight were used across these studies. Mean change in weight was positive in all studies. Mean LOS ranged widely between studies (Table 1).

**Table 1:** Studies of inpatient treatment for children and adolescent patients with anorexia nervosa

First Author, Year	# of Participants <18 yrs. (Diagnosis)	Study Design	Mean Length of Stay	Primary Outcome	Findings
*Studies of Inpatient Treatment Based on an Integrative Approach for AN*					
Anis, 2016 [18]	59 (47 AN-R^a^ and 12 atypical AN^b^)	Case series	67.2 days (SD=81.1 days)	Change in weight admission to discharge (BMI^c^)	Weight increased from mean BMI 16.3 (SD=1.35) to 17.1 (SD=1.35)
Ayton, 2009 [19]	50 (23 AN-R, 5 AN-B/P^d^ and 6 EDNOS^e^)	Case control (outcomes compared between those admitted with parental consent vs those formally detained)	240 days or 417 days	Change in weight admission to discharge (BMI)	Weight increased from BMI 14.4 (SD=1.9) to 18.5 (SD=1.6) or 16.6 (SD=2.6) to 19.6 (SD=1.5), difference in weight at admission between groups was significant (p=0.001) and non-significant at discharge.
Bourion-Bedes, 2013 [20]	66 (54 AN-R and 12 AN-B/P)	Case series	Not reported	Change in weight (BMI) and % of participants reaching 90%TGW^f^	Admission BMI 14.8 (SD=1.8) and change in BMI 3.1 (SD=1.4) (p<0.001) 90.9% of patients reached 90%TGW by a mean of 58.2 days (SD=38.3) of admission
Castro-Fornieles, 2007 [21]	49 (37 AN-R and 12 AN-B/P)	Case series	29.8 days (SD=17.6)	Change in weight admission to discharge (mean BMI), change mean EAT-26^g^ scores ANSOCQ^h^ scores admission to discharge	Weight increased from mean BMI 15.5 (SD=1.4) to 18.4 (SD=0.8) (p<0.001) Mean EAT-26 score decreased from 53.9 (SD=29.9) to 43.5 (SD=29.0) (p=0.002) Mean ANSOCQ score decreased from 53.6 (SD=19.7) to 62.9 (SD=24.5) (p=0.001)
Fennig, 2015 [36]	44 (all AN)	Case series	115.8 days (SD=54.7)	Change in weight admission to discharge (mean BMI), mean EDE-Q^i^ scores, mean EDI-2^j^ scores	Weight increased from mean BMI 16.2 (SD=1.8) at admission to 19.5 (SD=1.5) at discharge Change in total EDE-Q score was significant (p<0.05) No significant change in EDI-2 scores admission to discharge
Goddard, 2013 [22]	11 (AN or EDNOS)	Case Series	29.0 days (SD=11.9)	Change in weight admission to discharge (mean BMI), mean EDE-Q scores	Weight increased from BMI 15.0 (SD=1.6) at admission to 18.5 (SD=1.3) at discharge (p=0.001) EDE-Q total and subscales improved, but non-significant change admission to discharge
Heinberg, 2003 [23]	40 (27 AN-R, 8 AN-B/P, 2 “low weight” BN, 1 subthreshold AN-R, 1 subthreshold BN^k^, 1 atypical ED)	Case control (comparison to adult sample and inpatient to day treatment for adolescent)	20.6 days (SD=13.03)	Weight gain per week	Rate of inpatient weight gain mean 1.86kg/week (SD=0.68) vs adults gained 1.95 kg/week. Day Treatment weight gain 0.99kg/week (SD=0.47)
Hetman, 2017 [24]	51 (44 AN-R, 7 AN-B/P)	Case control (comparison of patients that did not require readmission by one year and those that did require re-admission)	112 days (SD=69.6) and 113.8 days (SD=34.8)	Weight gain admission to discharge (BMI) and Percent from Target Weight (PFTW) at Discharge, change mean EDE-Q scores admission to discharge	No readmission – increase in BMI from 16.4 (SD=1.8) to 19.6 (SD=1.1) vs readmission – increase in BMI from 16.39 (SD=2.0) to 19.89 (SD=1.19) PFTW in group not requiring readmission was 101.2% at discharge vs 98.2% in group requiring readmission (p<0.05) Change in EDE-Q scores did not differ between groups
Kalisvaart, 2007 [25]	39 (28 AN, 11 EDNOS)	Case Series	50.8 (SD=29.6)	Weight gain admission to discharge (%TGW and BMI) and rate of weight gain	%TGW rose from 74.8 (SD=9.4) at admission to 85.1 (SD=5.3) at discharge (p<0.001) BMI rose from a mean of 15.5 (SD=1.9) to 17.6 (SD=1.2) at discharge (p<0.001) Mean rate of weight gain was 0.1kg/d (SD=0.06)
Leon, 1985 [26]	31 (19 AN-R, 12 AN-B/P)	Case Control (AN-R vs AN-B/P)	143 (Range 56-276) for AN-R group, 135 (Range 43-242) for AN-B/P group	Weight gain (change in weight in kg admission to discharge)	Weight rose from a mean of 38.4kg (range 28.2-47.3) to 47.2kg (37.3-49.5) for AN-R group and from a mean of 42.0kg (35.0-48.6) to 46.8kg (34.1-54.5)
Lock, 2003 [37]	39 (all AN)	Case series	31.9 (SD=16.5)	Weight gain admission to discharge (%TGW)	Mean %TGW rose from 74.9% (SD=7.0%) at admission to 85.2% (SD=5.8%) at discharge
Lievers, 2009 [27]	268 (217 AN-R, 51 AN-B/P)	Case series	135 (SD=97)	Weight gain admission to discharge (BMI)	Mean BMI rose from 13.5 (SD=1.3) at admission to 17.4 (SD=1.4)
Mekori, 2017 [28]	88 (44 AN-R, 17 AN-B/P, 13 BN, 14 EDNOS-B/P)	Case control (compared patient with AN vs BN or EDNOS-B/P)	204 (SD=91)	Weight gain admission to discharge (BMI)	Mean BMI rose significantly from admission to discharge (15.8, SD=1.9 to 19.8, SD=0.7) for AN group.
Nova, 2008 [29]	14 (all AN)	Case Series	Unclear	Weight gain admission to one month (%TGW, weight in kg)	Mean %TGW rose from 72.2 (SD=7.9) at admission to 78.7 (SD=6.0) after one month (p<0.001) Mean weight at admission was 39.4 (SD=6.1) and rose to 43.0 (SD=4.9) after one month (p<0.001)
Rothschild-Yakar, 2011 [30]	62 (33 AN-R, 10 AN-B/P, 19 BN)	Case control (compared AN-R to AN-B/P and BN combined)	196.2 (SD=68.1) for AN-R group	Change in mean weight from admission to discharge (BMI) Change in EAT -26 scores admission to discharge	Mean BMI rose from 14.94 (SD=1.93) at admission to 19.24 (SD=1.45) at discharge for AN-R group. Mean EAT-26 score for AN-R group decreased from 41.8 (SD=18.6) to 32.2 (SD=22.2), ES (diagnosis x time = 0.23, NS)
Roux, 2016 [31]	83 (all AN)	Case control (compared adolescent to adult patients)	137.6 (SD=101.8)	Change in weight from admission to discharge (BMI)	Mean BMI rose from 14.3 (SD=1.44) at admission to 17.9 (SD=1.7) at discharge
Shugar, 1994 [32]	15 (all AN-B/P)	Case series	91 (range 84-98)	Change in weight admission to discharge (BMI) Change in EAT-26 score from admission to discharge	Mean BMI rose from 38.4 (SD=2.56) at admission to 16.3 (SD=0.98) at discharge Mean EAT-26 score decreased from 48.7 (SD=9.8) at admission to 29.6 (SD=9.7) at discharge
Tasaka, 2017 [33]	41 (all AN-R)	Case series	130.7 (SD=66.2)	Change in weight admission to discharge (BMI and %TGW)	Mean BMI rose from 13.2 (SD=1.6) at admission to 15.3 (SD=1.7) at discharge Mean %TGW rose from 68.3 (SD=7.3) at admission to 78.8 SD=3.6) at discharge
Toms, 1972 [34]	1 (AN)	Case Report	239	Change in weight admission to discharge (kg)	Weight increased from 25.5kg at admission to 42.6kg at discharge
Vall, 2017 [35]	40 (36 AN-R, 4 AN-B/P)	Case Series	20.1 (SD=10.3)	Change in weight admission to discharge (BMI centile) Change in EDE-Q score admission to discharge	Mean BMI centile rose from 9.0 (SD=2.1) at admission to 21.3 (SD=3.1) at discharge Mean total EDE-Q score did not differ significantly from admission to discharge
*Stuides Inpatient Treatment Utilizing a Family-Based or Cognitive Behavioural Approach for AN*					
Goldfield, 2003 [38]	1 (AN-R)	Case Report	Not Reported	Change in weight from admission to discharge (BMI)	BMI rose from 15.4 at admission to 19.5 at discharge.
Halvorsen, 2018 [39]	37 (all AN)	Case Series	144.2 (SD=95.2)	Mean change in weight during admission (kg)	Mean weight rose be 7.5kg (SD=4.4) during admission.
Matthews, 2016 [40]	1 (AN-R)	Case Report	9	Change in weight admission to discharge (BMI and %TGW)	BMI rose from 16.3 at admission to 17.5 at discharge %TGW rose from 82.0% at admission to 85.8% at discharge
Salbach-Andrae, 2009 [41]	57 (41 AN-R, 16 AN-B/P)	Case series	89.6 (SD=24.5)	Change in weight admission to discharge (BMI centile)	Mean BMI centile rose from 1.46 (SD=2.41) at admission to 9.44 (SD=6.68) at discharge
Schlegl, 2016 [42]	238 (188 AN-unspecified, 42 AN-B/P, 8 atypical AN)	Case series	81.9 (32.0)	Change in weight admission to discharge (BMI) Change in EDI-2 scores admission to discharge	Mean BMI rose from 14.8 at admission to 17.3 at discharge (ES=2.06) EDI-2 Global rose from 296.0 (SD=64.3) at admission to 245.7 (SD=65.1) at discharge (ES=0.78)
Paul, 2013 [43]	1 (AN-R)	Case report	6	Change in weight admission to discharge (kg)	Weight rose from 36.5kg at admission to 37.6kg at discharge
*Studies of Inpatient Treatment Utilizing a Behaviour Therapy Approach for AN*					
Alessi, 1989 [44]	1 (AN-R)	Case Report	70	Change in weight admission to discharge (kg) Mean EAT-26 scores admission to discharge	Weight rose from 23.8 kg at admission to 31.8kg at discharge EAT-26 scores remained high until 49 days into admission then decreased (actual absolute values not given)
Blanchet-Collet, 2016 [45]	1 (AN-R)	Case Report	Not Reported	Change in weight admission to discharge (BMI)	BMI rose from 13.5 at admission to 16.5 at discharge
Blinder, 1970 [46]	1 (AN-R)	Case Report	38	Change in weight admission to discharge (kg)	Weight rose from 42.6kg at admission to 51.0kg at discharge
Cinciripini, 1983 [47]	1 (AN-B/P)	Case Report	39	Change in weight admission to discharge (kg) Change in intake admission to discharge (kcal/day)	Weight rose from 44.0kg at admission to 54.0kg at discharge Daily intake rose from 850kcal at admission to 1700kcal at discharge
Clark, 1981 [48]	2 (AN-R)	Case reports	44 days and 56 days	Change in weight admission to discharge (kg)	Case 1: weight rose from 39.4kg at admission to 45kg at discharge Case 2: weight rose from 39kg to 50kg at discharge
Collins, 1983 [49]	37 (AN-R)	Case series	23.9 (range 2-66)	Weight gain admission to discharge (kg)	Mean weight gain was 5.8kg (range 0-12.7kg) from admission to discharge
Garfinkel, 1973 [50]	4 (AN-R)	Case reports	Case 1: 42days Case 2: 42 days Case 3: 56 days Case 4: 21 days	Weight gain while being treated under behavioural therapy framework (kg)	Case 1: 9.0kg Case 2: 8.6kg Case 3: 14.6kg Case 4: 11.9kg
Halmi, 1975 [51]	4 (all AN)	Case reports	Not specifically reported for adolescent patients, 43.8 days for adults and adolescents combined	Weight gain admission to discharge (%TGW) Total weight gain during admission (kg)	Mean %TGW at admission was 67.5% and at discharge was 84% Mean total weight gain during admission was 8.15kg
Leitenberg, 1968 [52]	2 (both AN)	Case reports	45 days and 39 days	Weight gain while treated under behavioural therapy framework (kg) Increase in intake while treated under behavioural therapy framework (kcal)	Case 1: weight rose 8.2 kg, intake rose from 1700kcal to 4000kcal Case 2: weight rose 11.2kg, intake rose from 1600kcal to 3900kcal
Nygaard, 1990 [53]	84 (all AN)	Case series	23 (range 7-56)	Weight gain admission to discharge (kg)	Mean weight gain from admission to discharge was 1.89 (SD=1.41)
Pertschuk, 1978 [54]	2 (both AN)	Case reports	13	Weight gain while treated under behavioural therapy framework (kg)	Case 1: 4.88 kg Case 2: 3.63 kg
Poole, 1978 [55]	2 (both AN)	Case reports	23	Weight gain while treated under behavioural therapy framework (kg)	Case 1: 12.6 kg Case 2: 15.3 kg
Solanto, 1994 [56]	53 (all AN)	Case control (compared 22 vs 31 patients treated under to different behaviour contracts)	28	Rate of weight gain when treated under each behaviour contract.	Group 1: 0.09kg/d (SD=0.05) Group 2: 0.16kg/d (SD=0.07) Significant effect for contract x day (p<0.009)
Steinhausen, 1985 [57]	24 (17 AN-R, 7 AN-B/P)	Case series	77	Weight gain admission to discharge (%TGW) Change in EAT-26 scores admission to discharge Change in EDI-drive for thinness admission to discharge	Mean weight rose from 65.9%TGW at admission to 87.4%TGW at discharge (p<0.0001) Mean EAT-26 score dropped from 37.1 to 12.7 (p<0.0001) Mean EDI-drive for thinness dropped from 8.0 to 1.9 (p<0.02)
*Studies of Inpatient Treatment Utilizing a Psychodynamic Approach for AN*					
Groen, 1966 [58]	5 (all AN)	Case reports	61 (range 30-91)	Weight gain admission to discharge (kg)	Mean weight rose from 36.6 kg (range 29-43kg) at admission to 43kg (range 36-47kg) at discharge
Kronenberg, 1994 [59]	1 (AN-R)	Case report	152	Weight gain admission to discharge (kg)	Weight rose from 35kg at admission to 42kg at discharge
*Studies of Inpatient Treatment on Pediatric Unit for AN*					
Jenkins, 1987 [60]	21 (16 AN-R, 5 AN-B/P)	Case series	185 (SD=122)	Weight gain admission to discharge (%TGW)	Mean %TGW rose from 68% (5.5) at admission to 99% (SD=7.7) at discharge
Maxmen, 1974 [61]	5 (all AN)	Case reports	40 (range 11-67)	Weight gain admission to discharge (kg)	Mean weight rose from 34.4kg at admission to 43.2 at discharge

^a^*AN-R* anorexia nervosa – restricting subtype

^b^*AN* anorexia nervosa

^c^*BMI* body mass index

^d^*AN-B/P* anorexia nervosa – binge/purge subtype

^e^*EDNOS* eating disorder not otherwise specified

^f^*TGW* treatment goal weight

^g^*EAT-26* eating attitude test

^h^*ANOSOCQ* anorexia nervosa stages of change questionnaire

^i^*EDE-Q* eating disorders examination - questionnaire

^j^*EDI-2* eating disorders inventory

^k^*BN* bulimia nervosa

Three studies (total *n* = 39) examined inpatient treatment utilizing a family-based approach [38–40], and three papers (total *n* = 296) reported on inpatient treatment utilizing a cognitive behavioural (CBT) approach [41–43]. In all studies patients gained weight in hospital, although measures by which weight changes were reported varied, as did LOS (Table 1).

Fourteen additional studies reported on a behaviour therapy approach (total *n* = 218) [44–57]. Various approaches to reporting change in weight were used, and all studies reported a positive change in weight from admission to discharge (Table 1). Of interest, Collins et al. (1983) reported that LOS was correlated to admission weight and feeding mode (i.e. oral vs nasogastric tube (NGT) feeds), and reported that the highest LOS was in those < 75% Treatment Goal Weight (TGW) at admission and requiring NGT feedings [49]. Another cohort study, by Solanto et al. (1994), reported weight gain under two types of behaviour contracts, varying only with regards to the expected rate of weight gain as measured every 4 days (q4d) (i.e. 0.36 kg/q4d vs 0.55 kg/q4d) [56]. Those treated under the contract with higher weight targets gained weight at a faster rate and gained more weight overall.

Two reports (total *n* = 6) were included in which patients were treated within an inpatient program using a psychodynamic approach [58, 59]. The LOS and weekly weight gain for these patients varied substantially (Table 1).

Finally, two reports examined the effect on weight of admission to a general pediatrics unit with supportive psychotherapy [37, 60, 61]. In both studies patients gained weight (Table 1).

#### Symptom change

While all of the observational studies of patients with AN using an integrative framework for inpatient treatment reported on change in weight, fewer reported on change in ED symptoms or psychopathology (Table 1). Four studies reported on changes in the Eating Disorder Examination-Questionnaire (EDE-Q) [22, 24, 35, 36]. In only one of these studies, Fennig et al’s (2015) [36], a significant pre-post difference (*n* = 51, *p* < 0.05) was found and attributed predominantly to changes in *restraint* and *eating concerns* subscales. Of note, BMI at discharge was higher in the Fennig et al. (2015) study than the other three studies that reported on EDE-Q scores. Three studies (total *n* = 126) reported change in Eating Attitude Test (EAT) scores at admission and discharge [21, 30, 32]. The difference in EAT scores was noted to improve in two of the studies [21, 32]. Fennig et al. (2015), compared scores on the Eating Disorder Inventory-2 (EDI-2) at admission and discharge and found no significant change in total or subscale scores [36].

Schlegl et al. (2016), used a CBT framework for treatment and reported on symptom change using EDI-2 scores [42]. All subscales showed significant improvements (Table 1).

Several studies using a behaviour therapy approach reported on symptom change during admission (Table 1). Cinciripini et al. (1983) described a case report in which purging after meals decreased from 48% of meals/week to 0% of meals per week [47]. Two other studies reported on EAT scores, both of which showed a reduction in scores during admission [44, 57]. Of note, Alessi et al. (1989) reported that EAT scores remained high for the first 7 weeks of treatment (with weight gain of 4.5 kg over the first 7 weeks) and scores then dropped (from total score of 60 to 10) during the last 3 weeks of a 10-week admission. Steinhausen et al. (1985) reported symptom change through EDI scores and noted that the mean “Drive for Thinness” scores decreased significantly over the course of admission (*p* = 0.02) [57].

#### Measures of change in motivation

Castro-Fornieles et al. (2007) measured motivation for change using the Anorexia Nervosa Stage of Change Questionnaire (ANSOCQ) at admission and discharge in an inpatient program utilizing an integrative framework [21]. Change in mean ANSOCQ score was noted to be statistically significant; however, both admission and discharge scores were characterized as within the “preparation” phase of motivation, and wide confidence intervals were reported (Table 1).

### Inpatient programs for mixed ED diagnoses

#### Weight gain

Three studies using an integrative approach (total *n* = 239) were included and reported on weight gain during inpatient treatment for patients with mixed ED diagnoses [9, 28, 30] (Table 2). The studies included in this section reported outcomes for various eating disorders (ie AN, BN, OSFED and EDNOS) as a combined group rather than differentiating outcomes by diagnosis. In all 3 studies patients gained weight from admission to discharge. Not surprisingly, in the 2 studies that differentiated anorexia nervosa-restricting subtype (AN-R) from other ED diagnoses, there was a significantly greater increase in Body Mass Index (BMI) for the group containing AN-R patients [28, 30].

**Table 2:** Studies of inpatient treatment for children and adolescents mixed eating disorder diagnoses

Author, Year	# of Participants (Diagnosis)	Study Design	Mean Length of Stay	Primary Outcome	Finding
Mekori, 2017 [28]	88 (44 AN-R, 17 AN-B/P, 13 BN, 14 EDNOS-B/P)	Case control (compared patient with AN vs BN or EDNOS-B/P)	204 (SD = 91)	Weight gain admission to discharge (BMI)	Mean BMI rose significantly from admission to discharge (15.8, SD^a^ =1.9 to 19.8, SD = 0.7) and did not increase significantly in the BN/EDNOS group (19.8, SD = 2.1 to 21.1, SD = 1.3); F (group x time) = 88.1, *p* < 0.0001)
Morris, 2015 [9]	89 (70 AN, 2 atypical AN, 1 BN, 16 Unspecified ED)	Case series	141.1 (SD = 125.7)	Weight gain admission to discharge (mean weekly weight gain and BMI)	Mean weekly weight gain was 0.43 kg/week and there was a significant rise in mean BMI admission to discharge (*p* < 0.001)
Rothschild-Yakar, 2011	62 (33 AN-R, 10 AN-B/P, 19 BN)	Case control (compared AN-R to AN-B/P and BN combined)	196.2 (SD = 68.1) for AN-R group, 178.8 (SD = 69) for AN-B/P and BN	Change in mean weight from admission to discharge (BMI) Change in EAT −26 scores admission to discharge	Mean BMI rose from 14.94 (SD = 1.93) at admission to 19.24 (SD = 1.45) at discharge for AN-R and from 18.80 (SD = 3.99) to 20.15 (SD = 2.29) for AN-B/P and BN. ES (diagnosis x time = 0.59, *p* < 0.001) Mean EAT-26 score decreased for AN-R group from 41.8 (SD = 18.6) to 32.2 (SD = 22.2) and in AN-B/P and BN group from 46.7 (SD = 15.0) to 28.8 (SD = 14.7). ES (diagnosis x time = 0.23, NS)

^a^*SD* Standard deviation

#### Symptom change

Rothschild-Yakar et al. (2013) compared symptom change from admission to discharge in an integrative inpatient treatment program for mixed ED diagnoses, using the EAT-26 in a group of patients with AN-R vs anorexia nervosa binge-purge subtype (AN-BP) or bulimia nervosa (BN) [30]. Overall there was a statistically significant improvement in EAT-26 scores over the course of the admission (*p* < 0.001), and no significant difference in change in EAT-26 scores by diagnosis.

### Inpatient programs for bulimia nervosa

We identified only one study that reported outcomes of inpatient treatment specifically for youth with BN [62]. The treatment provided was based on behaviour therapy and outcomes focused primarily on changes from admission to discharge in serotonin levels, as measured by 5-hydroxytriptamine induced calcium release from platelets. As such, this study was limited in that the only reported ED-specific outcome was change in BMI, and weight decreased slightly over admission (Table 3).

**Table 3:** Studies of inpatient treatment for children and adolescent with bulimia nervosa

Author, Year	# of Participants (Diagnosis)	Study Design	Mean Length of Stay	Primary Outcome	Finding
Wockel, 2009 [62]	13 (all BN)	Case series	69 (SD = 24.5)	Weight change admission to discharge (BMI)	Mean BMI decreased by 0.3 kg (SD=1.4) from admission to discharge

### Inpatient programs for ARFID

#### Weight

Three case reports or case series were identified which described inpatient treatment of a total of eight children with ARFID treated using either a family-based or CBT or behavioural therapy approach [63–65]. Weight gain was reported in two studies (Table 4).

**Table 4:** Studies of inpatient treatment for children and adolescents with avoidant restrictive food intake disorder

Author, Year	# of Participants (Diagnosis)	Study Design	Mean Length of Stay	Primary Outcome	Findings
Spettigue, 2018 [63]	3 (2 ARFID^a^-aversive subtype, 1 ARFID- mixed subtype)	Case reports	53 days	Change in weight gain (%TGW)	Case 1: Weight increased from 83%TGW to 100%TGW. Case 2: Weight increased from 75.8%TGW to 100%TGW. Case 3: Weight increased from 72%TGW to 88%TGW
Singer, 1992 [64]	3 (1 ARFID – aversive subtype, 2 ARFID-mixed subtype)	Case reports	32 days (range 16–60 days)	Change in weight (kg) Increase in caloric intake from admission to discharge (kcal/day)	Case 1: Increase in weight from 21.8 kg to 24.5 kg (over 60 days), intake increased from 1557 kcal/d to 2208 kcal/d) Case 2: Increase in weight from 21.4 kg to 22.6 kg (over 16 days), intake increased from 740 kcal/d to 1500 kcal/d) Case 3: Increase in weight from 17.7 kg to 18.0 kg (over 19 days), intake increased from 1200 kcal/d to 1500 kcal/d
Pitt, 2018 [65]	2 (ARFID – mixed subtype)	Case reports	Not reported	Reduction in vomiting and tolerance of oral intake without emesis	Vomiting frequency reduced and oral tolerance improved although specifics not reported

^a^*ARFID* Avoidant restrictive food intake disorder

#### Change in Oral intake

Singer et al. (1992) reported on change in caloric intake in kcal/day for three patients with ARFID using a CBT inpatient treatment [64]. Oral intake rose for all three patients over the course of admission (Table 4).

Pitt and Middleman (2018) reported on two cases of adolescents with ARFID [65]. After admission, nasojejunal (NJ) tubes were placed to initiate refeeding when oral feeding was not tolerated. The authors reported that the use of an individualized behaviour plan for each patient providing reinforcements for eating was the critical factor which helped these patients to tolerate oral intake without vomiting and allowed for the removal of the NJ tubes (Table 4).

### Combined inpatient and day treatment programs

#### Weight gain

Five studies summarized the experience of 264 adolescents with AN treated as inpatients followed immediately by day treatment (DTP), utilizing either an integrative or CBT approach [66–70]. Studies considered as providing combined inpatient and day treatment programs reported on change from the point of admission to an inpatient program through the end of their involvement in day treatment. In these studies, all patients received inpatient treatment directly followed by day treatment services. All five studies included inpatient treatment for medically unstable patients followed by transfer to a DTP once medical stability was attained (Table 5). Total mean LOS (i.e. inpatient and DTP combined) varied substantially between studies. Weight increased in all studies (Table 5).

**Table 5:** Studies of inpatient treatment followed by day treatment for children and adolescents with eating disorders

Author, Year	# of Participants (Diagnosis)	Study Design	Mean Length of Stay	Primary Outcome	Finding
Delle Grave, 2014 [66]	27 (all AN^b^, subtype not recorded)	Case series	91 days inpatient + 49 days day treatment	Weight gain from admission to inpatient to discharge from day treatment (kg, BMI centile) Change in EDE-Q^a^ scores from admission to inpatient until discharge from day treatment	Mean weight rose from 38.5 (SD = 6.1) at admission to 49.7 (SD = 5.6) at discharge Mean BMI centile rose from 2.7 (SD = 4.2) at admission to 34.2 (SD = 15.7) at discharge Mean EDE-Q Global score dropped from 3.7 (SD = 1.3) at admission to 2.1 (SD = 1.2) at discharge (*p* < 0.001)
El Ghoch, 2014 [67]	33 (all AN, subtype not recorded)	Case Control	91 days inpatient + 49 days day treatment	Weight gain admission to inpatient to discharge from day treatment (kg, BMI centile)	Mean weight rose from 38.9 kg (SD = 5.1) to 49.4 kg (SD = 3.9) at discharge (admit to discharge *p* < 0.001, admit vs control *p* < 0.001, discharge vs control *p* < 0.06) Mean BMI centile rose from 1.6 (SD = 4.0) at admission to 31.7 (SD = 10.6) at discharge (admit to discharge *p* < 0.001, admit vs control *p* < 0.001, discharge vs control *p* < 0.8)
Hillen, 2015 [68]	34 (31 AN-R^b^, 3 AN-B/P^b^)	Case series	105.7 (SD^c^=38.5)	Weight gain admission to inpatient to discharge from day treatment (BMI, %TGW^d^) Change in EDI-2^e^ scores admission to inpatient to discharge from day treatment Change in ANSOCQ^f^score admission to inpatient to discharge from day treatment	Mean BMI rose from 15.7 (SD = 1.2) at admission to 18.0 (1.0) at discharge Mean %TGW rose from 77.6% (SD = 5.4%) at admission to 88.5% (SD = 4.4%) at discharge Mean EDI-2 score dropped from 270.9 (SD = 66.6) at admission to 255.5 (SD = 78.1) at discharge (*p* < 0.36) Mean ANSOCQ score rose from 50.2 (SD = 14.8) at admission to 71.6 (SD = 23.0) at discharge (*p* < 0.0001)
Strober, 2006 [69]	99 (all AN, subtype not recorded)	Case series	118 (SD = 27)	Weight gain admission inpatient to discharge from day treatment (BMI)	Mean BMI rose from 12.5 (SD = 1.1) at admission to 18.5 (SD = 0.42) at discharge
Treat, 2008 [70]	71 (all AN, subtype not recorded)	Case series	33.6 days inpatient, 22.3 days day treatment	Weight gain admission to inpatient to discharge from day treatment (%TGW, BMI) Overall outcome as rated by composite of %TGW, weight trajectory and compensatory measures)	Mean %TGW rose from 74.1 (SD = 7.3) to 87.3% (SD = 5.77) at discharge (*p* < 0.01) Mean BMI rose form 15.2 (SD = 1.54) at admission to 17.9 (SD = 1.07) at discharge (*p* < 0.01) Excellent outcome in 25 patients, intermediate in 29 patients and poor in 17 patients at discharge from day treatment

^a^Eating Disorders Examination Questionnaire

^b^Anorexia Nervosa – binge/purge

^c^Standard Deviation

^d^Treatment Goal Weight

^e^Eating Disorders Inventory

^f^Anorexia Nervosa Stages of Change Questionnaire

#### Symptom change

Symptom change was reported using various scales in two studies of combined inpatient and DTP [66, 68]. Hillen et al. (2015) failed to demonstrate a significant change in EDI-2 scores. Dalle Grave et al. (2014) reported a significant decrease in EDE-Q scores pre to post treatment for global score and for all subscales other than Shape Concern. The percentage of patients with Global EDE-Q scores < 1 SD above the community mean at admission was 2% (+/− 7.7) and at discharge it was 10% (+/− 38.5), suggesting a decrease in ED thoughts and urges with treatment (Table 5).

#### Motivation

Hillen et al. (2015) reported on change in motivation as measured by the ANSOCQ in 35 patients [68]. Overall mean scores increased by 21.7 points which signified moving from contemplation to preparation phases. Overall, 29.4% (up from 0% at admission) of patients were classified as being in “maintenance phase” and 26.5% (up from 15% at admission) in “action phase” at time of discharge from DTP (Table 5).

#### Overall outcome

Treat et al. (2008) included 71 patients who combined inpatient and DTP and reported on “overall outcome” [70]. At the end of DTP 35.2% were deemed to have an excellent outcome, 26.8% were deemed to have good outcome,14.1% were deemed below average outcome and 23.9% were described as having a poor outcome, according to definitions assigned by the authors which included a combination of % ideal BMI, maintenance of weight and compensatory behaviours (Table 5).

### Adjunctive treatments

#### Adjunctive cognitive remediation therapy (CRT)

Four studies reported on the addition of CRT to integrative inpatient treatment for patients with AN (total *n* = 127) [71–74] (Table 6). Herbrich et al. (2017) described no difference in the change in weight between adolescent patients who received 10 sessions of CRT over 10 weeks compared to those who received treatment as usual (TAU) in a quasi-experimental design (*n* = 24 in each group) [74]. The other three studies did not include a control group. Patients (total *n* = 79) gained weight, but it was not possible to determine whether CRT had an impact on weight gain above and beyond what would have been expected by inpatient treatment [71–73].

**Table 6:** Studies of adjunctive treatments in addition to inpatient treatment for children and adolescents with eating disorders

Author, Year	# of Participants (Diagnosis)	Study Design	Mean Length of Intervention	Primary Outcome	Findings
Asch, 2014 [71]	2 (both AN-R)	Case reports	10 weeks	Change in weight (BMI pre/post) Change in EAT-26 score pre/post Change in EBRS^a^ score pre/post	Both patients gained weight (Pt 1: BMI 15.3 to BMI 18.1, Pt 2: BMI 13.3 to BMI 17.5) EAT-26 scores for both patients decreased over the intervention EBRS scores for both patients decreased over the intervention
Kuge, 2017 [72]	7 (all AN)	Case reports	4 weeks	Change in weight (BMI and %TGW pre/post)	Mean weight rose over the intervention (mean BMI rose from 14.8 to 16.0, ES^b^ 0.80 and mean %TGW rose from 73.6 to 79.4%, ES 0.68)
Harrison, 2017 [73]	70 (66 AN-R, 4 AN-B/P)	Case series	10 weeks	Change in weight (mean %TGW pre/post) Change in EDE-Q score pre/post Change in motivation (MSCARED^c^ pre/post)	Weight increased significantly during the intervention (79.3%TGW to 89.0%TGW, *p* < 0.001) Non-significant decrease in EDE-Q score (*p* < 0.08) Significant increase in motivational stage of change (*p* < 0.001)
Herbrich, 2017 [74]	48 (36 AN-R, 8 AN-B/P, 4 atypical AN)	Case control	5 weeks	Change in weight (mean BMI centile pre/post)	Significant increase in BMI centile in both groups pre/post (*p* < 0.006), no difference between groups
Depestele, 2017 [75]	112 (45 AN-R, 26 AN-B/P, 24 BN, 16 ED-NOS)	Case control (compared adjunctive multi-family group, n = 62 vs multi-parent group, n = 50)	8.5 weeks	Change in mean EDI-2 scores over time and between interventions Change in frequency of B/P behaviours over time	Significant improvement in mean EDI-2 subscales of drive for thinness (*p* < 0.001) and body dissatisfaction (*p* < 0.001). No significant difference between interventions BP behaviours decreased over time for patients with BN (*p* < 0.05) and AN-B/P (*p* < 0.01)
Janas-Kozik, 2011 [76]	24 (all AN-R)	RCT (inpatient bright light therapy + CBT,^d^ n = 12, vs inpatient CBT alone, n = 12)	6 weeks	Mean change in weight during intervention (BMI)	At end of intervention both groups had an increase in BMI of 10%, however significant increase from baseline in bright light group evident at week 3 vs week 6 for no bright light therapy
Couturier, 2009 [77]	21 (19 AN-R, 2 AN-B/P)	Case control (patients treated with meal support, n = 12, versus no meal support, *n* = 9)	61.2 (SD = 37.4) vs 78.0 (SD = 46.5), NS difference (*p* < 0.39)	Mean weekly weight gain Rate of need for NGT^e^ feeds	Mean weekly weight gain did not differ between groups (1.2 kg/week +/−1.0 vs 0.6 kg/week +/−0.4, *p* < 0.09) Patients receiving meal support had a significantly lower rate of NGT feeds than those without meal support (11.1% vs 66.7%, *p* < 0.02)
Kells, 2013 [78]	52 (restrictive ED, no specific diagnoses reported)	Case control (patients who received at least one supervised meal/admission, *n* = 13 vs those who received no meal support, n = 39)	8.4 (SD = 7.5) vs 5.9 (SD = 3.5), non-significant difference (*p* < 0.66)	Mean daily weight gain (kg)	No significant difference between groups (0.35 kg+/−0.23 kg/week vs 0.33 kg+/− 0.29 kg/week, *p* < 0.65)
Kells, 2016 [79]	108 (restrictive ED, no specific diagnoses reported)	Case control (patients with no meal support, *n* = 38 vs delayed meal support, *n* = 11 vs meal support, *n* = 54)	5.9 (SD = 3.5) vs 9.8 (SD = 7.3) vs 6.7 (SD = 3.3), non-significant difference (*p* < 0.27)	Mean daily weight gain (kg)	No significant difference between groups (0.34 +/−0.29 kg/d vs 0.31 +/− 0.26 kg/d vs 0.32 +/− 0.29 kg/d, *p* < 0.63)
Leacy, 2012 [80]	40 (38 AN-R, 2 AN-B/P)	Case control (patients treated with selective menus, n = 22, compared to non-selective menus, *n* = 18)	74.2 (SD = 28.7) vs 60.3 (SD = 22.8), non-significant difference (*p* < 0.09)	Mean weekly weight gain and change in EDE-Q scores	Patients treated with non-selective menus had a significantly higher weekly weight gain (0.95 +/− 0.35 kg/week) than those treated with selective menus (0.72 +/− 0.24 kg/week) (*p* < 0.02) There was no difference in change in EDE-Q scores between groups.

^a^*EBRS* Eating behaviors rating scale

^b^*ES* Effect size

^c^*MSCARED* Motivational stages of change for adolescents recovering from an eating disorder

^d^*CBT* Cognitive behavioral therapy

^e^*NGT* Nasogastric tube

Three studies of CRT added to inpatient treatment for AN reported on symptom change (Table 6). A report of two cases by Asch et al. (2014) described that total scores on the EAT decreased for one patient and increased in the other patient and Eating Behavior Rating Scale (EBRS) scores decreased slightly for both patients, by the end of 10 weeks [71]. Another study by Harrison et al. (2018), included 70 hospitalized patients who received individual CRT, and noted no change in EDE-Q scores [73].

In a study by Harrison et al. (2018), patients reported on change in motivation as measured by the MSCARED before and after the course of CRT [73]. There was a statistically significant shift in motivation noted (*p* < 0.001), with 42.9% in preparation, action or maintenance stages of change at initiation of CRT, and with 95.7% in one of those stages of change after receiving CRT (Table 6). Due to the design of this study it was not possible to differentiate the effect of inpatient treatment alone from inpatient treatment plus CRT.

#### Adjunctive multi-family/parent group therapy

A study by Depestele et al. (2017) reported on 112 patients with various ED diagnoses admitted to an inpatient ED unit providing an integrative therapy approach to treatment, who also received either adjunctive multi-family group therapy (MFT, *n* = 62) or adjunctive multi-parent group therapy (MPT, *n* = 50) [75]. Both MPT and MFT interventions “promoted an autonomy-supportive parental attitude and the adolescents’ autonomy and self-determination.” Results reported a main effect of time on drive for thinness (*p* < 0.001) and body dissatisfaction (*p* < 0.001) as measured by EDI-2. Both scales improved independent of the type of intervention (Table 6).

#### Adjunctive bright light therapy

A study by Janas-Kozik et al. (2011) examined patients with AN and depressive symptoms (> 17 on Hamilton Depression Rating Scale) who were admitted to a CBT-based inpatient program and treated adjunctively with Bright Light Therapy [76]. In this study patients were randomized to receive either daily 30 min of Bright Light Therapy (BLT) + inpatient treatment (*n* = 12) × 6 weeks, or inpatient treatment only × 6 weeks (n = 12). Patients in both groups had a significant change in their BMI during the 6-week study; however, weight change from baseline was statistically significant by week 3 (*p* = 0.038) in the BLT group but only at week 6 (*p* = 0.048) in the TAU group (Table 6).

#### Adjunctive meal support

Three studies examined the effect of meal support/supervision as part of inpatient treatment for groups of patients with mixed ED diagnoses [77–79] (Table 6). In the two studies examining weight gain, there were no significant differences between cohorts who received meal support (total *n* = 77) and those who did not (total *n* = 78) on the rate of weight gain per day, although there was a trend towards greater weight gain per day in the group who received meal support [78, 79]. In these two studies, the approach to meals for patients who did not receive meal support was not documented. A separate study by Couturier & Mahmood (2009) reported a significant decrease in the rate of nasogastric tube feeds in the cohort of patients treated on an inpatient unit after the institution of consistent meal support [77].

#### Selective versus non-selective menus

Leacy & Cane (2012) (*n* = 22) compared the rate of weight gain and EDE-Q scores in patients with AN who received non-selective menus (i.e. meals chosen by a dietician) or selective menus (i.e. meals chosen by the patient, under the direction of a dietician) as part of their inpatient treatment [80]. The non-selective menu group gained weight significantly faster than those in the selective menu group (*p* = 0.02) (Table 6). No significant differences were found on any of the EDE-Q items related to eating concern. Overall change in EDE eating concern scores in this study were low, ranging from − 0.6 to 1.1 (Table 6).

## Conclusions

The aim of the present review was to identify the scope and benefits of psychological treatment provided to children and adolescents with EDs in inpatient settings. We identified 66 studies that demonstrated considerable heterogeneity in the types of treatment provided and the outcomes reported. While all studies focused on inpatient treatment, in some studies this treatment was provided on a pediatric unit and in other studies it was provided on a psychiatric unit, either designed specifically for the treatment of patients with EDs or not. The treatment provided also varied with regards to the therapeutic modalities utilized, length of stay in intensive treatment, and the goals or expectations of treatment.

Despite the presence of substantial biases, and lack of control groups, each of the specialized ED inpatient programs and non-specialized general pediatric wards reported success in helping underweight patients with restrictive eating disorders gain weight. Beyond this finding, given the overwhelming heterogeneity of methods described, there is little that can be concluded from the various studies contained within.

Similarly, it is impossible to draw any conclusions as to which adjunctive treatments might be most helpful when treating youth with AN in hospital. While adjunctive treatments were examined, i.e. the use of meal support, selective versus non-selective menus, bright light therapy, CRT, and multi-family versus multi-parent group, the quality of the studies does not allow us to draw any conclusions about the effectiveness of these treatments. There are also some interesting omissions from this literature on adjunctive inpatient treatments, including no papers looking at outcomes of adjunctive yoga, exercise programs, art therapy, journaling, mindfulness, dialectical behaviour skills groups, or individual or group motivational enhancement therapy. Furthermore, there are insufficient studies of inpatient treatment for EDs other than AN, such that no conclusions can be drawn about the effectiveness of inpatient programs for the treatment of BN, ARFID or BED.

Finally, none of these studies serve to identify the specific factors most associated with a successful inpatient program for the treatment of children and adolescents with EDs. Clinical experience often points to a number of factors that appear to have clinical relevance, including a specialized, experienced multidisciplinary treatment team that combines confidence, compassion, consistency and the ability to create a safe environment where it is assumed that patients will take the nutrition they need to recover. These units typically provide skilled meal support and attend to patients’ medical, nutritional and psychological needs, with a focus on weight gain, renourishment and symptom interruption. It is widely recognized that families need to be supported and educated, and patients benefit from therapy which helps them to separate from and externalize the illness, thereby improving their motivation for recovery. Unfortunately, few of these factors have been identified, studied or discussed in the literature, there are no RCTs to provide guidance, and it is impossible to compare one inpatient program to another based on studies to date. It is also impossible to discern the optimal LOS, which combination of individual, group or family therapy is essential, or to determine the ideal group therapy content.

Limitations of this review include the inability to complete a meta-analysis or quantitative analysis due to the low quality and high heterogeneity of the studies available, the lack of inclusion of the search term “unspecified feeding and eating disorder” which could have yielded additional studies on this topic, a lack of studies specifically reporting on particular diagnoses including EDNOS, OSFED or atypical anorexia nervosa, and an inability to disaggregate findings based on subgroup such as gender or age (ie children vs adolescents).

Future research should focus on resolving these deficiencies through the use of studies based on high quality research methods, standardized operation of reporting treatment variables and outcomes, and multi-site designs to gather data on larger numbers of participants. Given the paucity of research in this field, the prolonged LOS (and associated costs) often observed, as well as the substantial variability by which treatments are delivered, it is critical that basic indicators of admission and treatment are standardized and reported, in order to continue to move the field forward. Only with improved research methods and reporting will it be possible to identify the key components of successful inpatient treatment and to improve outcomes for children and adolescents with EDs.

## Supplementary information

**Additional file 1.** Database Search Strategies.

## Data Availability

Data sharing is not applicable to this article as no datasets were generated or analysed during the current study.

## Abbreviations

AN: Anorexia nervosa
AN-R: Anorexia nervosa – restricting subtype
AN-B/P: Anorexia nervosa – binge/purge subtype
ANSOCQ: Anorexia nervosa stages of change questionnaire
ARFID: Avoidant restrictive food intake disorder
BED: Binge eating disorder
BLT: Bright light therapy
BMI: Body mass index
BN: Bulimia nervosa
CBT: Cognitive behaviour therapy
CRT: Cognitive remediation therapy
DTP: Day treatment program
EAT-26: Eating attitude test
ED: Eating disorder
EDE-Q: Eating disorder examination – questionnaire
EDI-2: Eating disorder inventory
EDNOS: Eating disorder not otherwise specified
GRADE: Grading of Recommendations, Assessment, Development and Evaluations
MPT: Multi-parent group therapy
MFT: Multi-family group therapy
NGT: Nasogastric tube
NJ: Nasojejunal
OSFED: Other specified feeding and eating disorder
PRISMA: Preferred Reporting Items for Systematic Reviews and Meta-analyses
RCT: Randomized control trial
TAU: Treatment as usual
