# Supplementary material for: Outcomes of inpatient psychological treatments for children and adolescents with eating disorders at time of discharge: a systematic review
Source: J Eat Disord. 2020 Jul 3;8:32. doi: 10.1186/s40337-020-00307-2 (PMC7333407; doi:10.1186/s40337-020-00307-2)
Supplement: Supplementary file 1 — Additional file 1. Database Search Strategies. [file 40337_2020_307_MOESM1_ESM.docx]

Additional File 1.Database Search Strategies.

Database: MEDLINE <1950 to March 3, 2017> (4017 citations)

“eating disorders” [All fields] OR “anorexia nervosa”[All fields] OR “bulimia nervosa”[All fields] OR “eating disorder not otherwise specified”[All fields] OR “Other specified Feeding and eating disorder” [All fields] OR “avoidant/restrictive food intake disorder”[All fields] AND “inpatient”[All fields] OR “day treatment” [All fields] OR “residential” [All fields] OR “outpatient” [All fields]

Database: EMBASE <1974 to March 3, 2017> (4604 citations)

“eating disorders” [All fields] OR “anorexia nervosa”[All fields] OR “bulimia nervosa”[All fields] OR “eating disorder not otherwise specified”[All fields] OR “Other specified Feeding and eating disorder” [All fields] OR “avoidant/restrictive food intake disorder”[All fields] AND “inpatient”[All fields] OR “day treatment” [All fields] OR “residential” [All fields] OR “outpatient” [All fields]

Database: Cochrane Central Register of Controlled Trials (CENTRAL) <1996 to March 3, 2017> (56 citations)

“eating disorders” [All fields] OR “anorexia nervosa”[All fields] OR “bulimia nervosa”[All fields] OR “eating disorder not otherwise specified”[All fields] OR “Other specified Feeding and eating disorder” [All fields] OR “avoidant/restrictive food intake disorder”[All fields] AND “inpatient”[All fields] OR “day treatment” [All fields] OR “residential” [All fields] OR “outpatient” [All fields]

Database: Cochrane Reviews <1996 to March 3, 2017> (270 citations)

“eating disorders” [All fields] OR “anorexia nervosa”[All fields] OR “bulimia nervosa”[All fields] OR “eating disorder not otherwise specified”[All fields] OR “Other specified Feeding and eating disorder” [All fields] OR “avoidant/restrictive food intake disorder”[All fields] AND “inpatient”[All fields] OR “day treatment” [All fields] OR “residential” [All fields] OR “outpatient” [All fields]

Database: CINAHL <1984 to March 9, 2017> (797 citations)

“eating disorders” [All fields] OR “anorexia nervosa”[All fields] OR “bulimia nervosa”[All fields] OR “eating disorder not otherwise specified”[All fields] OR “Other specified Feeding and eating disorder” [All fields] OR “avoidant/restrictive food intake disorder”[All fields] AND “inpatient”[All fields] OR “day treatment” [All fields] OR “residential” [All fields] OR “outpatient” [All fields]

Database: PSYCHINFO <1967 to March 9, 2017> (2588 citations)

“eating disorders” [All fields] OR “anorexia nervosa”[MeSH Terms] OR “bulimia nervosa”[All fields] OR “eating disorder not otherwise specified”[All fields] OR “Other specified Feeding and eating disorder” [All fields] OR “avoidant/restrictive food intake disorder”[All fields] AND “inpatient”[All fields] OR “day treatment” [All fields] OR “residential” [All fields] OR “outpatient” [All fields]
